# Mammalian birth versus arousal from hibernation: thyroid hormones, common regulators of metabolic transition?

**DOI:** 10.1007/s00360-025-01611-6

**Published:** 2025-04-10

**Authors:** Melanie Heidkamp, Annika Herwig, Dominique Singer

**Affiliations:** 1 Division of Neonatology and Pediatric Critical Care Medicine, Center for Obestrics and Pediatrics, University Medical Center Hamburg-Eppendorf, Martinistrasse 52, 20246 Hamburg, Germany; 2 Institute of Neurobiology, Ulm University, Ulm, Germany

**Keywords:** Mammals, Hibernation, Torpor, Arousal, Birth, Thyroid

## Abstract

Mammalian birth and arousal from hibernation are both endogenously regulated transitional events, characterized by an increase in metabolic rate (MR) and onset of thermogenesis. Thyroid hormones (THs) are known to be key regulators of metabolic and thermogenic activity. To explore the similarities and differences in the role of THs during mammalian birth as opposed to arousal from hibernation, a comprehensive review is given of the levels and kinetics of serum thyrotropin-releasing hormone (TRH), thyroid stimulating hormone (TSH), thyroxine (T_4_), triiodothyronine (T_3_), and reverse triiodothyronine (rT_3_) in hibernating mammals upon arousal and in mammalian neonates at birth. The results for arousal are more heterogeneous than those for birth, reflecting different hibernation patterns between species as well as varying sampling times and methods. Overall, serum TRH concentrations were found to be decreased, TSH unchanged, and T_4_, T_3_, and rT_3_ mostly increased. In contrast, the data for mammalian birth show a marked increase in serum levels of TRH, TSH, T_4_, and T_3_, particularly in human neonates, with inconsistent results for rT_3_. In conclusion, both during arousal from hibernation and mammalian birth, THs play a critical yet not exclusive role in metabolic transition. In hibernators, the metabolic effects of THs appear to be mediated by the conversion rates in target tissues rather than by their serum levels alone, suggesting a sustained readiness for arousal. This contrasts with mammalian newborns, who at the beginning of their autonomous life experience the first activation of their thyroid gland, resulting in a transitory “hyperthyroid” state.

**Supplementary Information:**

The online version contains supplementary material available at 10.1007/s00360-025-01611-6.

## Introduction

### Phenomenological and physiological similarities of arousing hibernators and newborn mammals

Torpor and hibernation, characterized by a transient and endogenously controlled depression of metabolic rate (MR), body temperature (T_*b*_) and reduced physical activity, are omnipresent survival strategies in temperate zone animals to endure adverse environmental conditions and, in some cases, to avoid predation (Melvin and Andrews 2009; Wang and Wolowyk 1988; Ruf and Bieber 2023). Typically, natural forms of metabolic suppression require a process of transition: The hibernator transforms from a life with high metabolic activity to a life on a low flame, with reduced T_*b*_ during the inactive period of hibernation (Heldmaier 2011). This inactive hibernation period is repeatedly interrupted by periodic arousals, called interbout arousals (IBAs), and is terminated by a final arousal that ends the hibernating season (Epperson and Martin 2002; Ju et al. 2011). When arousing, the animal rapidly re-ignites intrinsically downregulated metabolic functions, elevates T_*b*_ and switches to its active state (Lust et al. 1989).

Most intriguingly, just as a hibernating animal rewarms and arouses from its metabolic suppression, a newborn increases its MR at birth and initiates thermoregulatory mechanisms to maintain an adequate T_*b*_. The fetus grows and matures in an environment of low oxygen partial pressure (*p*O_2_) during the intrauterine period and shows a reduced MR for its body size compared to the postnatal period. The intrauterine life is dependent on a functioning placenta to facilitate gas exchange, fetal circulation, and waste management as well as to provide a nutritional connection (Doherty et al. 2021). The deprivation of placental function after cord clamping therefore requires a drastic physiological adaptation of the newborn.

Hence, both phenomena – the arousal from hibernation as well as the event of birth itself – depict a unique and endogenously regulated transition process: The torpid animal revives from the depression of metabolic action to an active state of life, showing physiological similarities to a newborn adapting from a dependent, passive incubation in the well-temperate surroundings of the maternal womb to an autonomous life (Singer 2023).

**Fig. 1:**
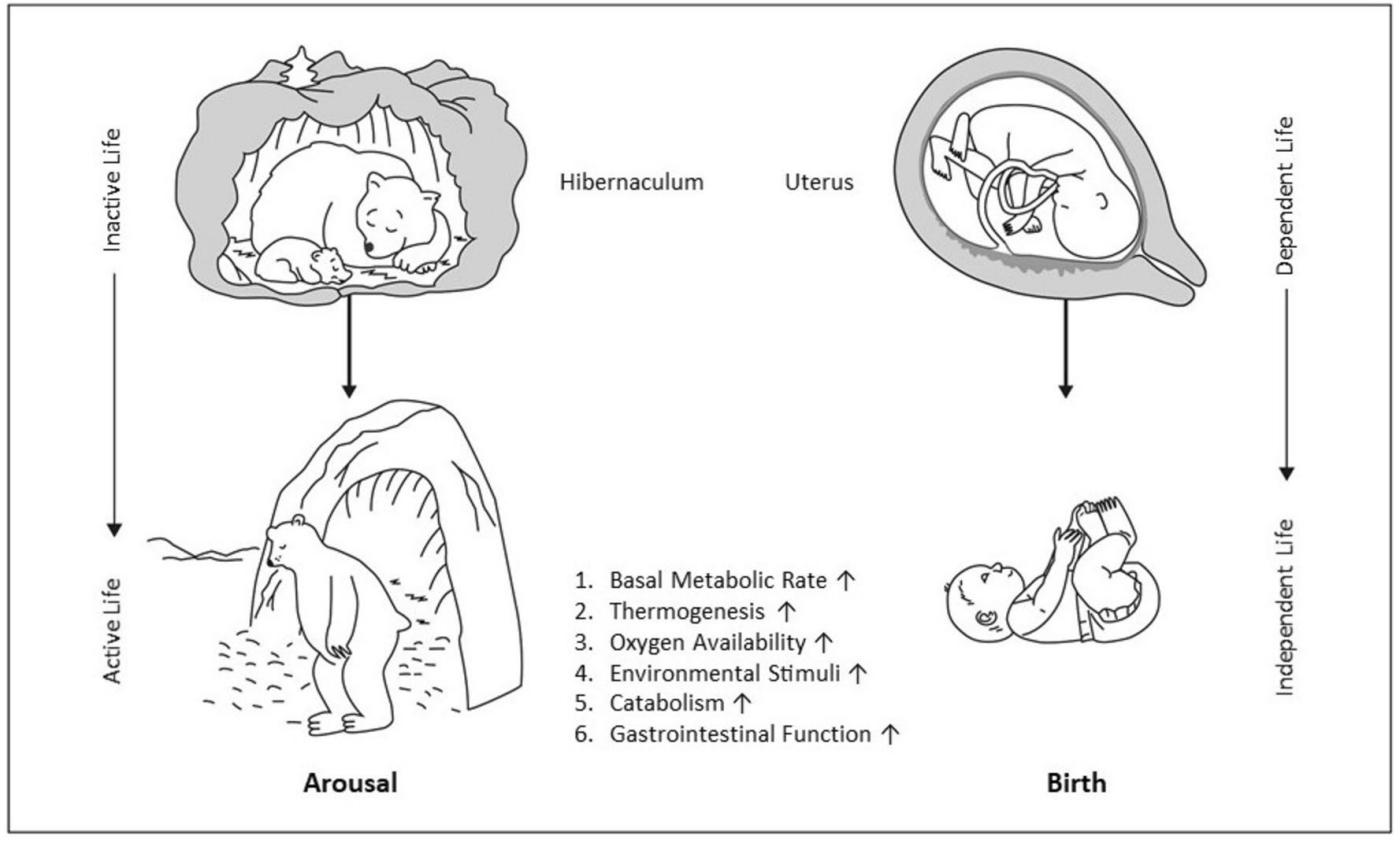
Phenomenological and physiological parallels between arousal from hibernation and mammalian birth

**Fig. 2:**
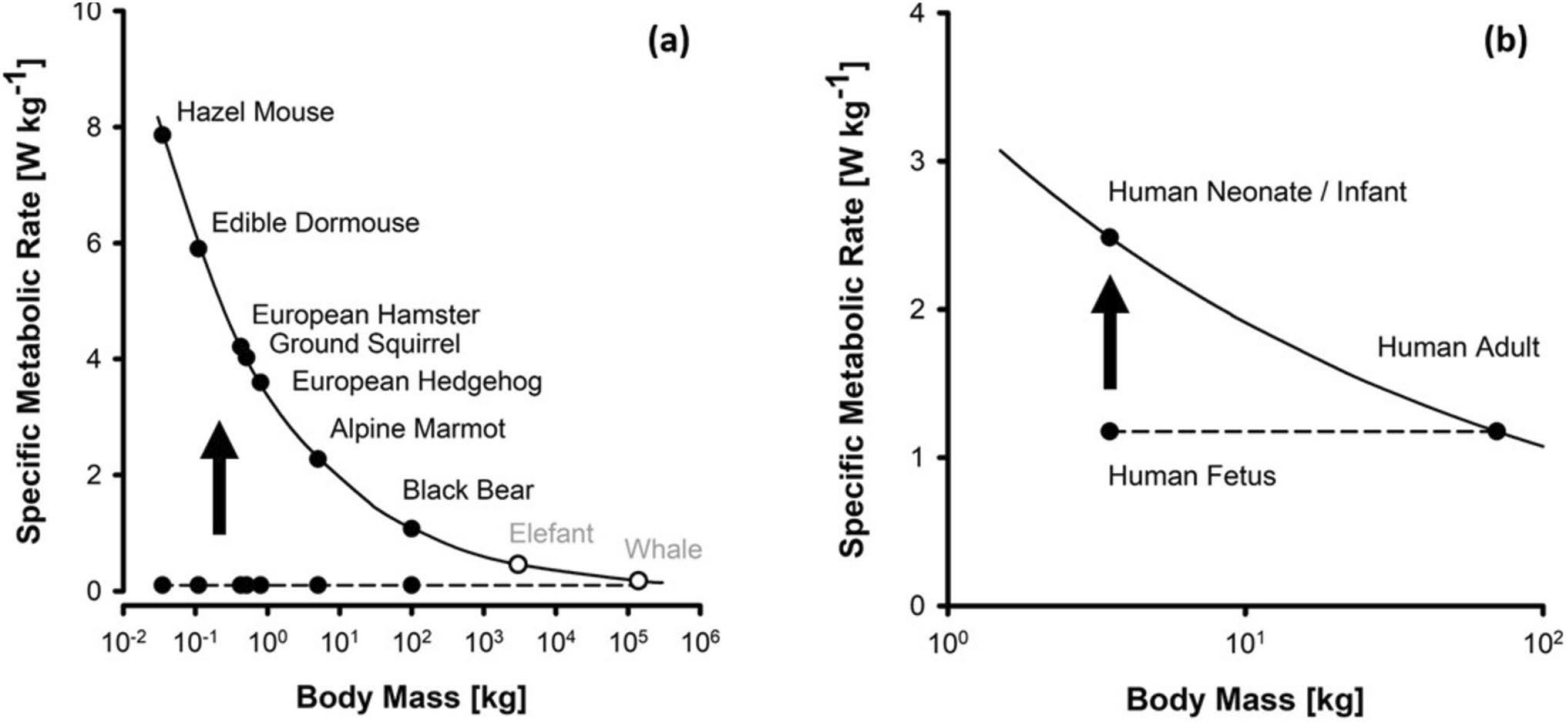
Deviation from the overall metabolic size relationship (Kleiber’s rule) in hibernating mammals (**a**) and mammalian (human) fetuses (**b**). Deeply hibernating individuals of several mammalian species exhibit a fairly uniform minimal specific metabolic rate, corresponding to the specific basal metabolic rate achieved by the very largest mammals due to their body size alone. While in the womb, mammalian fetuses behave more or less “like organs of the mother”, with the metabolic increase to the level expected from body size occurring after birth. The latter parallels the “switching on” of the overall metabolic size relationship taking place upon arousal from hibernation (redrawn from Singer 2004, 2023)

Alongside the analogy in activity, both transition processes share even more astounding parallels (Fig. 1):

- During hibernation and in utero, hibernator and fetus both deviate from the specific (mass-related) basal MR expected from their body size (Singer 2004, 2023) (Fig. 2a, b). This mathematical relation between MR and body mass (= metabolic size allometry), also known as Kleiber’s rule (Kleiber 1961), states that small mammals usually express higher specific MRs (in Watts per kg body mass) to compensate for the greater heat loss due to their large surface-to-volume ratio (White and Seymour 2005; Rubner 1883). The hibernating animal as much as the fetus exhibit an inappropriately low specific MR during intrauterine life and hibernation (Heldmaier et al. 2004; Singer 2004). During arousal the hibernator is able to endogenously increase its MR rapidly (Regan et al. 2019). Similarly, during birth a quick rise of the neonatal MR “up to the level to be expected from body size” ends the neonatal deviation from metabolic size allometry (Singer and Mühlfeld 2007). Intriguingly, a systematic deviation from this overall metabolic rule has been exclusively seen during the perinatal and perihibernating periods as yet.
- During the hibernating period, animals temporarily decrease thermogenesis to minimize energy output. The transition to higher metabolic activity in the phase of arousal requires the reactivation of thermogenesis to achieve an euthermic state. The neccessary heat is mainly generated by non-shivering thermogenesis (NST) in the brown adipose tissue (BAT) (Cannon and Nedergaard 2004). The fetus also shows suppressed thermogenesis in utero (Asakura 2004). Unlike mammals that hibernate at low ambient temperatures (T_*a*_), the fetus behaves like an estivating animal that reduces MR to maintain a constant T_*b*_ at high T_*a*_ (Grimpo et al. 2013). On exiting the uterine incubator, the newborn must stabilize T_*b*_ on its own using facultative thermogenic mechanisms in BAT as well (Graves and Haley 2013).
- During hibernation and torpor the animal reduces heart and respiratory rates significantly (Lust et al. 1989; Milsom and Jackson 2011; Lyman and Chatfield 1955; Zimmer and Milsom 2002). The extent of reduction varies and depends on differing hibernating patterns of the species. Seasonal hibernators usually mark the low end of the spectrum, they can drastically decrease breathing rates and exhibit low O_2_ availability (McArthur and Milsom 1991; Zimmer and Milsom 2002; Ruf and Geiser 2015; Heldmaier and Ruf 1992; Wilz and Heldmaier 2000). During the arousal period, concomitant to the increase in MR, a restoration of ventilation and heart rate and an increase in O_2_ availability can be observed (Heldmaier et al. 2004; Milsom and Jackson 2011; Andrews 2007). The animal switches over from low to high O_2_ consumption (Heldmaier et al. 2004; Milsom and Jackson 2011; Andrews 2007). Analogously, the fetus grows in a low-oxygen environment–resembling the *p*O_2_ levels on Mount Everest–compared to postnatal surroundings (Singer and Mühlfeld 2007; Morton 2016). Aborning, the neonate commences breathing on its own for the first time, in an environment of elevated O_2_ concentration when contrasted with its intrauterine condition increasing overall O_2_ availability (Graves and Haley 2013).
- During the inactive period, the hibernator rests in a hibernaculum whereas the fetus grows and matures in the maternal uterus. Hibernaculum and uterus constitute environments with little visual, auditive, olfactory, and tactile stimuli (Heldmaier et al. 2004; Boron and Boulpaep 2017). Additionally, both shelters aim to provide a more or less constant T_*a*_, thus minimizing undulation in thermal stimuli. During final arousal and birth the hibernator and the newborn are confronted with many surging environmental stimuli (Boron and Boulpaep 2017).
- Arousal and birth both implicate energetically expensive processes that lead to a quick depletion of energy resources in the blood stream of hibernator and fetus requiring alternate energy sources (Graves and Haley 2013; Heldmaier et al. 2004). Catabolic reactions serve the purpose to provide cells with quickly available energy substrates to fuel energetic needs. Arousing hibernator and neonate show an activation of catabolic reactions such as hepatic gluconeogenesis leading to increased glucose availability for cellular functions (Graves and Haley 2013; Carey et al. 2003).
- Upon arousal at the end of the hibernating season, fat-storing hibernating animals that do not feed throughout the entire hibernating season upregulate their previously unused gastrointestinal function and their gut physiology is being remodeled (Hume et al. 2002). During the intrauterine period, the fetus is supplied with nutrition via the placental connection, thus not requiring gastrointestinal activity (Graves and Haley 2013). As the newborn loses its nutritional link with the placenta at birth, the infant must rely on its own gastrointestinal tract (Graves and Haley 2013). In the first 48 h postnatally, a human neonate usually passes meconium, its first stool formation, indicating activation of the gastrointestinal tract (Graves and Haley 2013; Jerdee et al. 2015).

### Thyroid hormones as regulators of metabolic activity

**Fig. 3:**
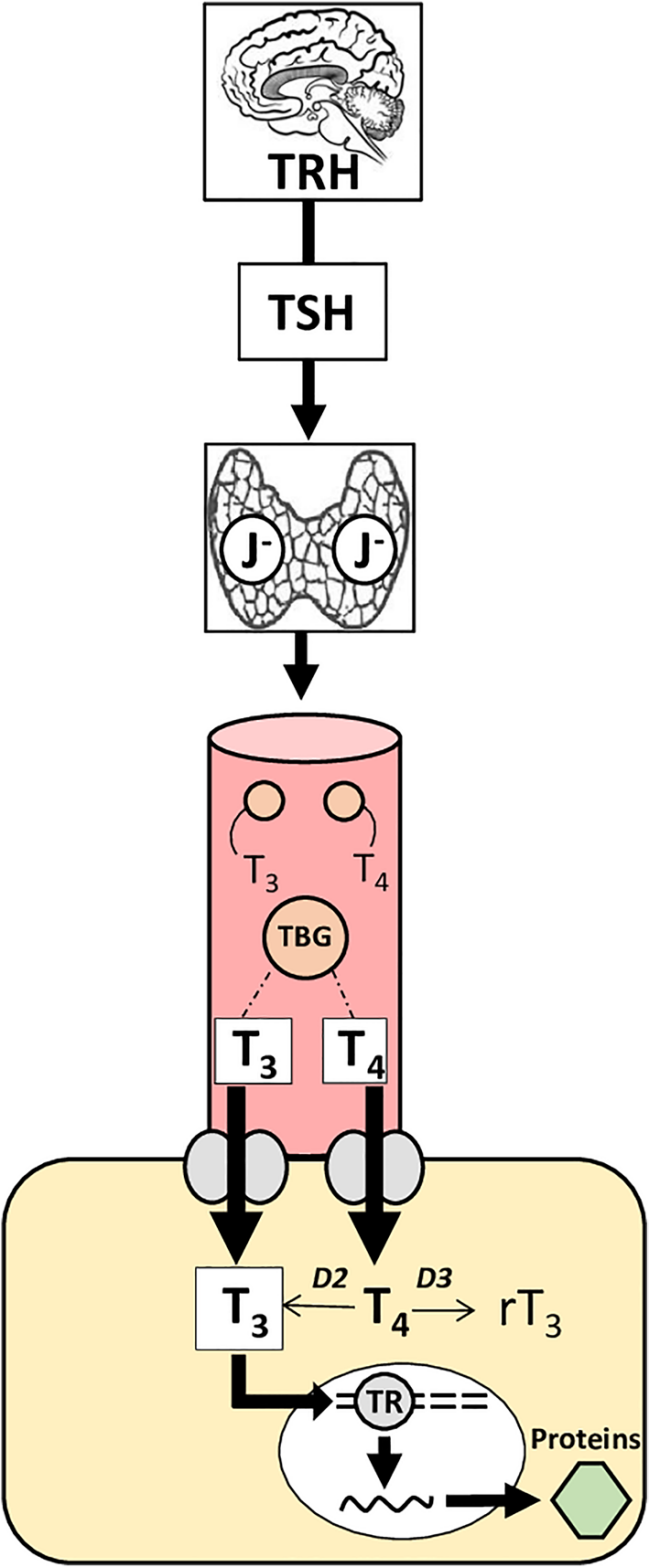
The Hypothalamus-Pituitary-Thyroid (HPT) axis. Thyroid-Releasing Hormone (TRH) and Thyroid-Stimulating Hormone (TSH) control the iodine (J^–^) dependent synthesis and release of thyroid hormones (THs; T_4_ = Thyroxine and T_3_ = Triiodothyronine) into the bloodstream. Within the bloodstream THs can be bound to Thyroid Hormone Binding Globulins (TBG) as well as circulate freely. Membrane receptors and transporters determine cellular TH uptake. Intracellular deiodinases (D2, D3) lead to either active (conversion of T_4_ to T_3_) or inactive (rT_3_ = reverse Triiodothyronine) metabolites. Activated nuclear thyroid hormone receptors (TRs) modify gene expression and, thus, cytoplasmic protein synthesis

For a long time, thyroid hormones (THs), thyroxine (T_4_) and triiodothyronine (T_3_), have been known to be key regulators of metabolic activity. They are produced and secreted by the thyroid gland which is controlled by the hypothalamus-pituitary-thyroid-axis (HPT-axis) (van der Spek et al. 2017) (Fig. 3). Thyrotropin-releasing-hormone (TRH), synthesized in the paraventricular nucleus of the hypothalamus, stimulates the pituitary gland to release thyroid stimulating hormone (TSH) which, in turn, triggers the thyroid gland to secrete T_4_ and T_3_ into the vascular system (Ortiga-Carvalho et al. 2016). In the vascular system THs can either be bound to thyroid hormone binding globulins (TBG) or circulate freely (Little 2016). Freely circulating THs in the blood stream are available for intracellular uptake depending on the availability of cellular TH transporters and membrane receptors that are target cell-specific (Visser 2000; Little 2016). Once transported across the plasma membrane, major pathways of TH metabolism, such as (a) deiodination, (b) sulfation, (c) glucuronidation, and (d) ether-link cleavage, further determine TH bioavailability and action within target cells (van der Spek et al. 2017). These metabolic pathways lead to either inactive or active TH metabolites and are controlled by the expression of specific enzymes in the target tissue itself (van der Spek et al. 2017). THs can exert their effects directly in the cytosol as well as in the cellular nucleus of target cells by T_3_ (Cheng et al. 2010). T_3_ is considered to be biologically more active than T_4_ (Gereben et al. 2008) and binds to nuclear thyroid hormone receptors (TRs) that function as transcription factors which can modify gene expression within hours (Yen 2001; Mullur et al. 2014).

The role of TH action in arousal from hibernation and torpor as well as in mammalian birth has been a popular subject of research for decades. If THs are involved in these transition processes, a change in hormone concentration should be expected. The aim of this review is (a) to create a pattern on the dynamics of TH concentrations and (b) to outline the role of THs during the metabolic transition of arousal from hibernation and mammalian birth.

## Materials and methods

This comprehensive literature review compiles the existing data on the dynamics of TRH, TSH, T_4_, T_3_, and rT_3_ concentrations in mammalian neonates, including human infants, at birth and in hibernating mammals upon final arousal. Publications including information on circulating TRH, TSH, T_4_, T_3_, and rT_3_ levels in plasma of human and mammalian neonates at the time of birth and of mammalian hibernators around the time of arousal from hibernation were collected on the data basis PubMed Central by using the following search terms: „*thyroid hormones and birth*“, „*thyroid hormone axis and birth*“, „*thyroid hormone system and birth*“, „*TSH and birth*“, „*TRH and birth*“ and „*thyroid hormones and arousal from hibernation*“, „*thyroid hormone axis and arousal from hibernation*“, „*thyroid hormone system and arousal from hibernation*“, „*TSH and arousal from hibernation*“, „*TRH and arousal from hibernation*“ as well as „*thyroid hormones and arousal from torpor*“, „*thyroid hormone axis and arousal from torpor*“, „*thyroid hormone system and arousal from torpor*“, „*TSH and arousal from torpor*“, „*TRH and arousal from torpor*“, „*thyroid hormones and metabolic suppression*“, „*thyroid hormone axis and metabolic suppression*“, „*thyroid hormone system and metabolic suppression*“, „*TSH and metabolic suppression*“, „*TRH and metabolic suppression*“. To maximize the identification of suitable studies, a manual search based on the reference list of studies was added.

All publications that contained information on changes of TRH, TSH, T_4_, T_3_, and rT_3_ concentrations of mammalian neonates around the time of birth and of mammalian hibernators surrounding the arousal process in the full text version in English language were included. Publications that incorporated only one absolute value of a single measurement were excluded for the reason of impossible value dynamic assessment from single numbers. This refers largely to publications containing suggested reference values for postnatal TH concentrations in the human newborn with regard to ethnicity and sex. Accordingly, only publications that allowed an evaluation of concentration dynamics – categorized as (a) increased, (b) unchanged, and (c) decreased – were included.

A complete reference to all sources cited in the tables, as far as they are not mentioned in the paper itself, is provided in the supplementary material.

## Results

### General characteristics of the retrieved data sources

The included studies contain information of mostly total and free T_4_ and T_3_ concentrations, a few exclusively quantified total T_4_ and T_3_ concentrations (e.g., Stubbe et al. 1978; Similä et al. 1975). Different methods of quantification of serum TRH, TSH, T_4_, T_3_, and rT_3_ levels were used with the radioimmunoassay (RIA) representing by far the most preferred technique, followed by electrochemiluminescence immunoassays (e.g., Mutlu et al. 2012), bioassays (e.g., Yamazaki et al. 1961), the Sephadex Column Method using commercial kits (e.g., Rogowski et al. 1974; Pezzino et al. 1981), and the dual-channel well counter and double-isotope counting procedure (e.g., Nelson et al. 1973).

The number of examined hibernators per study was generally smaller than the number of included newborns at birth. The timespan of the publication dates varies from 1968 to 2018 for arousal from hibernation or torpor and from 1958 to 2022 for mammalian birth. The quantity of publications pertaining information of TRH, TSH, T_4_, T_3_, and rT_3_ dynamics at birth outnumbers the available information regarding mammalian hibernators at interbout and final arousals. The number of studies that include measurements of TRH, TSH, T_4_, T_3_, and rT_3_ levels for the final arousal process are scarce, so inclusion criteria for the time frame of TH measurement were widened. Also data obtained at the end of the hibernation season was included in order to gain an impression or tendency of TRH, TSH, T_4_, T_3_, and rT_3_ dynamics at best for the period surrounding the arousal process.

### Dynamics of thyroid hormone concentrations in mammalian hibernators upon arousal

Publications containing information for TRH, TSH, T_4_, T_3_, and rT_3_ level dynamics in hibernators mostly determined values during the active period contrasted with the hibernating period (e.g., McCain et al. 2013) or collected data before and after hibernation (e.g., Nelson et al. 1973). Research on the dynamics of TH concentrations upon final arousal has been conducted on mammalian hibernators such as bears, hamsters, squirrels, woodchucks, echidnas, and bats (Table 1).

**Table 1:** Compilation of references on the dynamics of TRH, TSH, T_4_, T_3_, and rT_3_ concentrations in mammalian hibernators upon (final) arousal

		References
TRH	Decreased	Young et al. 1979b (woodchuck)
TSH	Unchanged	Bauman et al. 1968 (hamster)
T_4_	Increased	Azizi et al. 1979 (black bear), Damassa et al. 1995 (bats), Demeneix and Henderson 1978a, 1978b (squirrels), Hulbert and Hudson 1976 (squirrels), Kwiecinski et al. 1991 (bats), Magnus and Henderson 1988a (squirrels), Nevretdinova et al. 1992 (squirrels), Tomasi et al. 1998 (black bear), Wilsterman et al. 2015 (squirrels), Young et al. 1979a (woodchuck)
	Unchanged	Bauman et al. 1968 (hamster), Magnus and Henderson 1988a (squirrels), McCain et al. 2013 (black bear), Nelson 1973 (black bear), Nelson et al. 1973 (bear)
T_3_	Increased	Azizi et al. 1979 (black bear), Damassa et al. 1995 (bats), Demeneix and Henderson 1978a, Demeneix and Henderson 1978b (squirrels), Hulbert and Hudson 1976 (squirrels), Magnus and Henderson 1988a, 1988b (squirrels), Nevretdinova et al. 1992 (squirrels), Nicol et al. 2000 (echidnas), Tomasi et al. 1998 (black bear), Young et al. 1979a (woodchuck)
	Unchanged	Bauman et al. 1968 (hamster), Magnus and Henderson 1988a (squirrels), McCain et al. 2013 (black bear), Nelson 1973 (black bear), Nelson et al. 1973 (bear), Richardson et al. 2018 (bats)
rT_3_	Increased	Azizi et al. 1979 (black bear)

TRH levels at the time of arousal have been measured in woodchucks and found to be decreased (Young et al. 1979b). TSH concentrations have been described to not significantly change within the arousing hamster, *Mesocricetus auratus* (Bauman et al. 1968). T_4_ and T_3_ were seen to increase at the end of hibernation and upon arousal (10 studies for T_4_ and 9 studies for T_3_) or to remain unchanged (5 studies for T_4_ and 6 studies for T_3_). The values for rT_3_ have been observed to rise upon arousal in the black bear (1 study). Differences in the onset, extent, and velocity of TH dynamics around the time of arousal have been noticed according to species, sex, and individuals.

### Dynamics of thyroid hormone concentrations in mammalian neonates at birth

#### Human neonates

The available studies for all neonates, including human newborns, usually compared values in cord blood to concentrations measured either in hourly (e.g., Hüfner et al. 1973; Pezzino et al. 1981) or in daily intervals (e.g., Mutlu et al. 2012) after birth (Table 2).

**Table 2:** Compilation of references on the dynamics of TRH, TSH, T_4_, T_3_, and rT_3_ concentrations in human neonates

		References
TRH	Increased	Lombardi et al. 1978
TSH	Increased	Abuid et al. 1974, Cavallo et al. 1978, Chen et al. 2024, Czernichow et al. 1971, Fisher and Odell 1969, Fisher et al. 1969, Fisher et al. 1976, Fisher et al. 2000, Geiger 1973, Homoki et al. 1975, Hüfner et al. 1973, Jacobsen et al. 1977, Klein et al. 1982, Knobel 2007, Kratzsch and Pulzer 2008, Lemarchand-Béraud et al. 1972, Lombardi et al. 1978, Mutlu et al. 2012, Oddie et al. 1978, Odell et al. 1967, Pezzino et al. 1981, Polak and Luton 2014, Rogowski et al. 1974, Sack et al. 1976, Similä et al. 1975, Stubbe et al. 1978, Utiger et al. 1968
	Unchanged	Yamazaki et al. 1961
T_4_	Increased	Abuid et al. 1973, Abuid et al. 1974, Cavallo et al. 1978, Cavallo et al. 1980, Chen et al. 2024, Czernichow et al. 1971, Erenberg et al. 1974, Fisher and Odell 1969, Fisher et al. 1969, Fisher et al. 1976, Fisher et al. 2000, Jacobsen et al. 1977, Klein et al. 1982, Knobel 2007, Kratzsch and Pulzer 2008, Lemarchand-Béraud et al. 1972, Lombardi et al. 1978, Montalvo et al. 1973, Mutlu et al. 2012, Oddie et al. 1978, Pezzino et al. 1981, Pickering et al. 1958, Polak and Luton 2014, Rogowski et al. 1974, Santini et al. 1999, Similä et al. 1975, Stubbe et al. 1978, Touzery et al. 1978
	Unchanged	Hüfner et al. 1973
T_3_	Increased	Abuid et al. 1973, Abuid et al. 1974, Cavallo et al. 1978, Cavallo et al. 1980, Czernichow et al. 1971, Erenberg et al. 1974, Fisher et al. 1976, Jacobsen et al. 1977, Klein et al. 1982, Knobel 2007, Lombardi et al. 1978, Montalvo et al. 1973, Mutlu et al. 2012, Oddie et al. 1978, Pezzino et al. 1981, Polak and Luton 2014, Santini et al. 1999, Similä et al. 1975, Stubbe et al. 1978
	Unchanged	Rogowski et al. 1974
	Decreased	Chen et al. 2024, Hüfner et al. 1973, Kratzsch and Pulzer 2008
rT_3_	Increased	Cavallo et al. 1980, Chopra 1974, Santini et al. 1999
	Unchanged	Chopra et al. 1975
	Decreased	Kratzsch and Pulzer 2008

Plasma TRH levels were measured at birth and in 30 min time intervals up to 48 h post-partum by radioimmunoassay and found to peak at delivery up to 30 min followed by a rapid decrease reaching normal TRH concentrations at 24 h post-partum (Lombardi et al. 1978). At birth, increased TSH values have been documented by several authors. These early RIA studies revealed a surge in TSH secretion in the human newborn (Odell et al. 1967; Utiger et al. 1968). Some early studies using bioassay techniques measured no gradient in TSH concentration between maternal and cord blood (Costa et al. 1965; Yamazaki et al. 1961; Fisher et al. 1969) presumably due to „the lesser precision and sensitivity of the bioassay as contrasted with the radioimmunoassay“ (Fisher et al. 1969). More recent studies using electrochemiluminescence immunoassays were able to reproduce the results of elevated TSH concentrations in the newborn when compared to adults (Mutlu et al. 2012). One study measured higher TSH serum levels in Caucasian neonates compared to other ethnic groups (van Eekelen and Stokvis-Brantsma 1995). A physiological “hyperthyroid” state of the human newborn within the first days of postnatal life has been subject to many investigations. Levels of T_4_ and T_3_ were shown to increase (27 studies for T_4_ and 19 studies for T_3_), to remain within euthyroid ranges (2 studies) and to remain low following birth (2 studies for T_3_). A positive correlation to the gestational age of the newborn has been described (Bernard et al. 1977; Cuestas 1978; Fisher 1975; Oddie et al. 1977; Wilson et al. 1982; Mathur et al. 1986). Values of rT_3_ increased (3 studies), persisted (1 study) or decreased postnatally (1 study). Differences in the extent of increase, especially for T_3_ and T_4_, have been observed depending on gestational age (Jacobsen et al. 1977; Cuestas 1978; Erenberg et al. 1974; Uhrmann et al. 1978), sex (Tenore et al. 1980; Aktas et al. 2017), mode of delivery (Aktas et al. 2017), ethnic groups (Kratzsch et al. 2008; Boucai and Surks 2009; van Eekelen and Stokvis-Brantsma 1995), and location and timing of blood collection (Jacobsen and Peitersen 1979; Aktas et al. 2017) as well as differences between individuals (van Eekelen and Stokvis-Brantsma 1995).

#### Non-human mammals at birth

Research on the TRH, TSH, T_4_, T_3_, and rT_3_ dynamics of perinatal levels of non-human mammals at birth was mainly conducted on rats, calves, foals, lambs, piglets, and seals (Table 3).

**Table 3:** Compilation of references on the dynamics of TRH, TSH, T_4_, T_3_, and rT_3_ concentrations in non-human mammals at birth

		References
TRH	Increased	Dussault and Labrie 1975 (rats)
TSH	Increased	Cabello and Wrutniak 1990 (lambs), Dussault and Labrie 1975 (rats), Fisher et al. 1976 (lambs), Kieffer et al. 1976 (rats), Nathanielsz 1975 (calves, lambs), Sack et al. 1976 (lambs), Slebodziński and Cogiel 1983 (piglets)
T_4_	Increased	Cabello and Levieux 1980 (lambs), Cabello and Wrutniak 1986 (lambs), Cabello and Wrutniak 1989 (cattle), Cabello and Wrutniak 1990 (lamb), Davicco et al. 1982b (calves), Davicco et al. 1982a (lambs), Engelhardt and Ferguson 1980 (seals), Fisher et al. 1976 (lambs), Grünberg et al. 1998 (calves), Habibu 2022 (goat), Haulena et al. 1998 (seals), Hernandez et al. 1972 (calves), Irvine and Evans 1975 (foals), Kahl et al. 1977 (calves), Kieffer et al. 1976 (rats), Klein et al. 1980 (lambs), Leatherland 1979 (seals), Little 1991 (seals), Nathanielsz 1969 (lambs), Nathanielsz and Thomas 1973 (calves), Nathanielsz 1975 (calves, lambs), Nowak 1983 (piglets), Pals et al. 1973 (guinea pig), Sack et al. 1976 (lambs), Slebodziński 1971 (piglets, calves, lambs), Slebodziński and Cogiel 1983 (piglets), Slebodziński et al. 1981 (piglets), Brzezińska-Slebodzińska and Slebodziński 1986 (piglets), Steinhardt et al. 1996 (calves), Stokkan et al. 1995 (seals), Takahashi et al. 2001 (calves), Woldstad and Jenssen 1999 (seals), Wrutniak and Cabello 1987 (lambs)
	Unchanged	Davicco et al. 1982b (calves), Klein et al. 1978 (lambs), Kühn et al. 1986 (lambs)
	Decreased, low	Dussault and Labrie 1975 (rats), Klein et al. 1978 (lambs), Nathanielsz 1969 (lambs)
T_3_	Increased	Cabello and Levieux 1980 (lambs), Cabello and Levieux 1981 (lambs), Cabello and Wrutniak 1986 (lambs), Cabello and Wrutniak 1990 (lambs), Cabello and Wrutniak 1989 (cattle), Davicco et al. 1982b (calves), Davicco et al. 1982a (lambs), Dussault and Labrie 1975 (rats), Engelhardt and Ferguson 1980 (seals), Fisher et al. 1976 (lambs), Grünberg et al. 1998 (calves), Haulena et al. 1998 (seals), Irvine and Evans 1975 (foals), Kahl et al. 1977 (calves), Klein et al. 1980 (lambs), Klein et al. 1980 (lambs), Leatherland 1979 (seals), Little 1991 (seals), Mathur et al. 1980 (lambs), Nathanielsz 1969 (lambs), Nathanielsz et al. 1973 (lambs), Nathanielsz and Thomas 1973 (calves), Nathanielsz 1975 (calves, lambs), Nowak 1983 (piglets), Pals et al. 1973 (guinea piglets), Sack et al. 1976 (lambs), Slebodziński 1971, Slebodziński 1971 (calves, lambs), Slebodziński et al. 1981 (piglets), Slebodziński and Cogiel 1983 (piglets), Brzezińska-Slebodzińska and Slebodziński 1986 (piglets), Steinhardt et al. 1996 (calves), Stokkan et al. 1995 (seals), Takahashi et al. 2001 (calves), Woldstad and Jenssen 1999 (seals), Wrutniak and Cabello 1987 (lambs)
	Unchanged	Davicco et al. 1982b (calves), Habibu 2022 (goat), Kühn et al. 1986 (lambs)
rT_3_	Increased	Klein et al. 1980 (lambs), Haulena et al. 1998 (seals), Nowak 1983 (piglets), Slebodziński et al. 1981 (piglets), Brzezińska-Slebodzińska and Slebodziński 1986 (piglets), Wrutniak and Cabello 1987 (lambs)
	Unchanged	Klein et al. 1978 (lambs)
	Decreased	Cabello and Wrutniak 1986 (lambs), Cabello and Wrutniak 1990 (lambs), Klein et al. 1978 (lambs), Kühn et al. 1986 (lambs), Mathur et al. 1980 (lambs)

TRH concentrations have been shown to increase in rats in the postnatal period as well as their TSH values, although with a marked delay of 10–12 days (Dussault and Labrie 1975). A more brisk TSH surge at the time of birth has been described for lambs, calves, and piglets. Levels for T_4_ and T_3_ in mammalian newborns have been reported to increase (32 studies for T_4_ and 36 studies for T_3_), to remain unchanged compared to their fetal values (3 studies for T_4_ and 2 studies for T_3_) and to decrease shortly after birth (3 studies for T_4_). Differences in the onset, extent, and speed of TH augmentation at birth between species and breeds (Davicco et al. 1982; Grünberg et al. 1998), gestational age (Erenberg 1978; Uhrmann et al. 1978; Jacobsen et al. 1977; Sakaguchi et al. 1983), birth weight (Cabello and Levieux 1980), sex (higher in females) (Grünberg et al. 1998; Steinhardt et al. 1996) as well as individual differences (Grünberg et al. 1998), e.g., „lamb-to-lamb variation “ (Eales and Small 1986), have been observed (Slebodziński et al. 1981). One study found differing concentrations depending on the sampling location, i.e. for total T_4_ and free T_4_ between cord blood (higher) and jugular blood (lower) in foals taken one hour postpartum „whereas total T_3_ and free T_3_ were markedly lower in cord blood than in jugular blood taken later" (Irvine and Evans 1975). Values of rT_3_ increased in lambs, piglets, and seals (Klein et al. 1980; Nowak 1983; Slebodziński et al. 1981; Wrutniak and Cabello 1987; Haulena et al. 1998), persisted in lambs (Klein et al. 1978) or decreased in lambs postnatally (Cabello and Wrutniak 1986; Kühn et al. 1986; Mathur et al. 1980).

## Discussion

When analyzing the changes in thyroid hormone status at birth, as opposed to those occurring upon arousal from hibernation and torpor, a distinction must be made between blood concentrations and bioavailability. In addition, the considerable heterogeneity resulting from different hibernating patterns and varying degrees of maturity at birth has to be taken into account. Finally, the regulatory, or co-regulatory, effects in the target tissues need to be considered, bearing in mind the fundamental differences between the two transitional events as well as their obvious parallels.

### Dynamics of peripheral thyroid hormone concentrations upon arousal and at birth

#### Inconsistent findings upon arousal from mammalian hibernation

The arousal process at the end of the hibernating season implies a drastic change of metabolic and thermogenic activity as well as a re-ignition of previously decelerated physiological functions such as heart and respiratory rate. The importance of a thyroid gland for hibernators upon arousal that ended the hibernating season was suggested in early thyroidectomy studies when the absence of a thyroid gland prevented this process altogether (Patton and Platner 1971; Smith and Hoijer 1962). Seasonal variations in the morphology of the thyroid gland have already been described for several hibernating mammals, including ground squirrels, dormice, and hedgehogs, early on (Nevretdinova et al. 1992; Azzali et al. 1990; Alia 1957), to the point of a morphological quiescence of the thyroid gland during hibernation with signs of reactivation at the end of hibernation and the onset of arousal (Kayser 1961; Hoffman et al. 1965). Prior to arousal, while still hibernating, an increase in I-131 uptake has been seen in species like the woodchuck, the dormouse, the ground squirrel, and the golden hamster (Wenberg and Holland 1972) concluding that the thyroid gland produces THs before arousal (Lachiver 1952; Vidovic and Popovic 1954; Knigge et al. 1957; Yousef et al. 1967; Olivereau 1970).

**Fig. 4:**
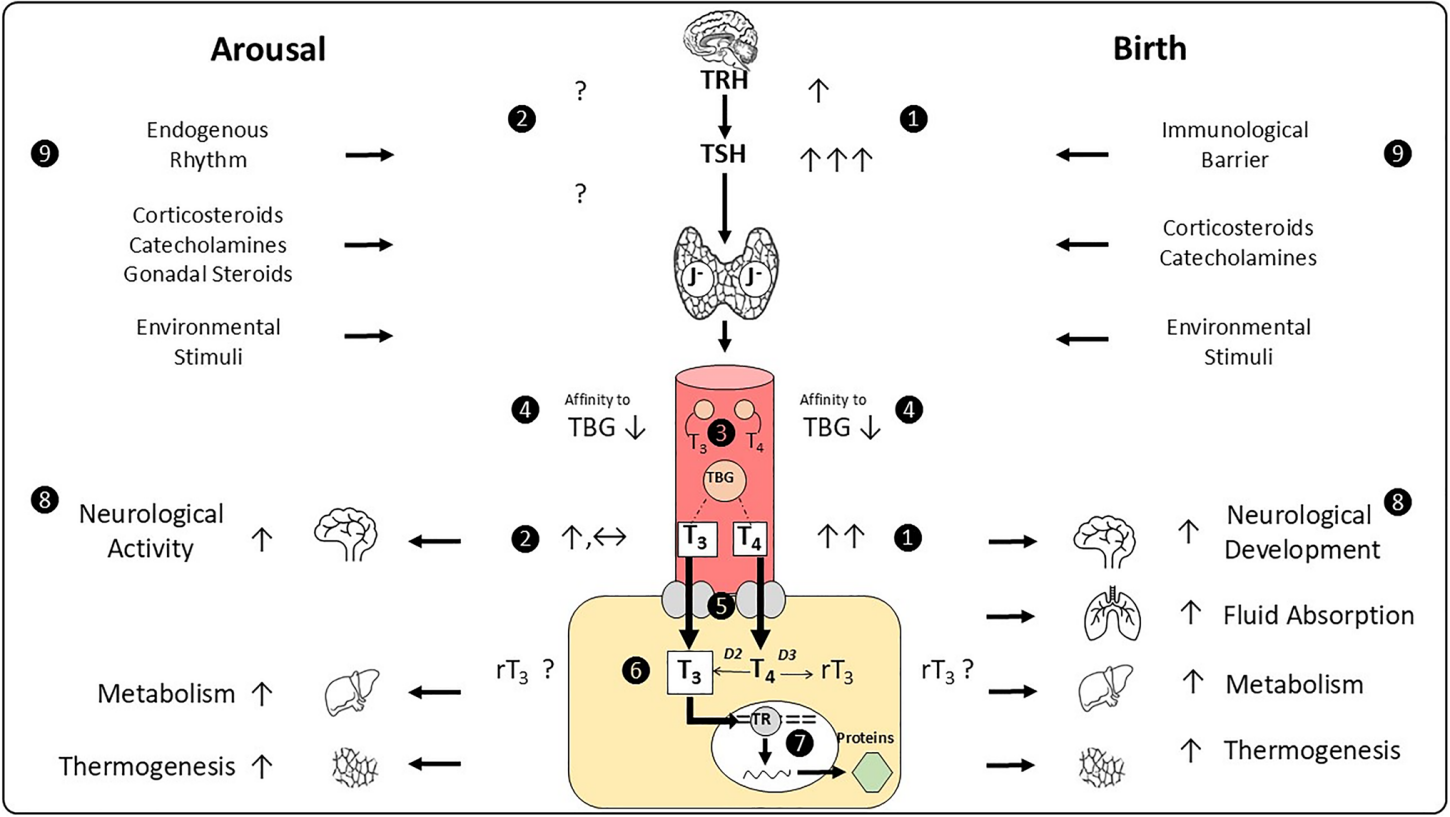
Role of the HPT axis in arousal from hibernation compared to mammalian birth. The circled numbers refer to the corresponding labels in the text. In both cases, THs are part of an interplay of endocrine stimuli to increase basal MR, thermogenesis, and neural activity. However, whereas the initial activation of the thyroid gland at birth results in a transitory “hyperthyroidism” as a start to autonomous life, the temporary silencing of TH activity in hibernation is more likely to be regulated at the target tissue level. Here, the continued presence of THs appears to ensure a sustained readiness for arousal

The collected available data on plasma TRH, TSH, and TH concentrations in mammalian hibernators around the time of arousal is heterogenous. The results do not indicate a distinct trend of serum TH concentration (Fig. 4, ②). Moreover, measurements are not as readily reproducible, sometimes contradictory. For example, a study of Azizi et al. showed significantly increased T_3_ and T_4_ levels in the black bear upon arousal time (Azizi et al. 1979) (total THs measured by RIA; free THs measured by equilibrium dialysis) whereas an investigation of McCain et al. registered no distinct pattern in their measured (total and free) TH concentrations when black bears terminated the hibernating season (McCain et al. 2013). Further, another study could not confirm morphological changes in the thyroid gland of the bear throughout the season that has been described for other hibernating mammals (Nelson et al. 1973). And Chmura et al. demonstrated an activation of the retrograde TSH/Deiodinase/T_3_ signaling pathway while arctic ground squirrels were still hibernating and deprived of photic or other environmental stimuli (Chmura et al. 2022).

#### Impact of differing hibernating patterns

One reason for these inconsistent findings may be that hibernators differ in their degree of metabolic reduction. Some species draw their energy from fat and some from food stores (Geiser and Ruf 1995). Some species hibernate for an entire season that can last weeks to months with little or no interruption (Geiser and Ruf 1995). This kind of *seasonal* hibernation generally requires (more) preparational procedures, the frequency of interruption through IBAs is low and ends with a final arousal that terminates the hibernating season (Blanco et al. 2018). Other animals show repeated episodes of reduced activity (torpor bouts) for up to 24 h, interspersed with higher frequencies of IBAs as periods of increased activity. This type of daily torpor can occasionally be induced with an amazing degree of flexibility (Blanco et al. 2018). This disparity – with smooth transitions in reality – implicates variations in metabolic preparational procedures (Hudson 1981; Rothwell 1982) around the time of arousal, e.g., endocrine, and therefore presumably resulting in differing findings according to hibernation preferences.

#### Transient state of “physiological hyperthyroidism” at mammalian birth

The major transition from dependent, intrauterine to an autonomous, extrauterine life happening at mammalian birth involves an adaptation in metabolic and thermogenic activity as well as an initiation of various physiological functions such as the pulmonary function. THs are known to support this transition already prenatally, for instance by promoting the maturation of fetal lungs (Barker et al. 1988, 1990) and the heart muscle (Forhead and Fowden 2014). The compiled data is more homogenous than for arousing hibernators. It illustrates a picture of a transient state of “physiological hyperthyroidism” with increased TSH, T_4_, and T_3_ concentrations during the postnatal period of the human and the mammalian, non-hibernating newborn (Fig. 4, ①). Additionally, TH fluctuations and increase are much more pronounced when compared to the arousal process. This endocrine TSH, T_4_, and T_3_ surge surrounding the process of being born has been known as one of numerous endocrine preliminaries that are required in order to accomplish this adaptation process (Hillman et al. 2012; Forhead and Fowden 2014).

#### Impact of differing maturity at birth

Analogously to differing hibernating patterns, the degree of neonatal maturity at birth differs in mammalian newborns considerably. The dichotomy to describe the two extremes of neonatal development in literature differentiates between *altricial* and *precocial* newborns (Derrickson 1992; Ferner et al. 2017). Precocial neonates–like hoofed mammals, dolphins, whales, elephants, and bats (Martin 2007)–are characterized by a relatively quick development into independent individuals that can survive on their own (Ferner et al. 2017). Altricial offspring, on the other hand–such as rats (Lamers et al. 1986) and carnivores–are reliant on parental care for a certain period following birth. Human newborns are–even though descendant of precocial relatives –functionally altricial since they are born helplessly and thus largely dependent on their caretakers to survive (Slonecker 2017). Precociality in eutherian neonates usually implies the most advanced organ development enabling to maintain vital functions, stabilize T_*b*_, and increase metabolism (Ferner et al. 2017). The high degree of neonatal maturity suggests that these neonates could be better equipped to adapt to extrauterine conditions (Ferner et al. 2017). However, whether it implies a difference in the endocrine preparation, like a more pronounced state of perinatal hyperthyroidism, remains to be elucidated.

### Regulation of peripheral thyroid hormone activity upon arousal and at birth

#### Comparative regulation of bioavailability in the bloodstream

Assuming that THs are involved in both adaptation processes, an increase of hormone concentrations upon transition time should become apparent. Yet, the analysis of plasma concentrations generally represents a momentary state of circulating THs in the blood and does not reflect the underlying dynamics of TH variations. It rather illustrates a current share of the total TH serum volume permitting increased metabolic activity, depending on developmental stages and environmental circumstances (Little 2016). Serum proteins bind most of TH (Yen 2001) and can further add to the freely circulating fraction of TH available for uptake by target tissues (Fig. 4, ③) (van der Spek et al. 2017). Several investigators noticed that the reduction of T_*b*_ during torpor causes an apparent increase in serum binding affinity for TH (Magnus and Henderson 1988a), and, inversely, suggested that increased T_*b*_ results in higher free T_4_ and T_3_ serum concentrations because of a reduced binding affinity (Fig. 4, ④) (Magnus and Henderson 1988a; Hudson 1981; Rothwell 1982). This would in turn lead to an increased amount of THs without the thyroid gland having to become more active (Rothwell 1982; Hudson 1981) during IBAs, for instance (Young et al. 1979a). The observation that reduced binding affinity to serum binding proteins can augment the fraction of free serum THs was described by several authors not only for hibernators (Galster and Morrison 1966; Magnus and Henderson 1988a) but also mammalian newborns like lambs and foals (Fig. 4, ④) (Cabello and Wrutniak 1990; Irvine and Evans 1975; Grünberg et al. 1998).

Consequently, free THs represent a regulatory mechanism to briefly enhance the TH action in order to meet increasing energetic demands. Practically, this could mean two things: (a) Serum proteins serve as a TH storage unit (van der Spek et al. 2017) that can be quickly depleted upon demand depending on the environmental stimulus, e.g., during brief arousal bouts or at birth when thermogenic effort is considerably amplified, and, (b) the amount of freely circulating THs permits rapid metabolic fluctuations when needed.

#### Comparative regulation of bioavailability in target tissues

Bioavailability of THs in target tissues can strongly deviate from measured free-plasma levels, depending on the expression of membrane transporters permitting intracellular uptake of T_4_ and T_3_ (Fig. 4, ⑤) (van der Spek et al. 2017). So far, it is unknown whether cells of effector organs can be sensitized or sensitize themselves for augmented TH uptake on demand during the arousal or birth process, for example by a sudden and quick upregulation in translation of TH membrane transporters. The possibility of cell-specific responses on demand, however, has been suggested in the woodchuck that showed varying responses of individual thyroid cells to acute TSH stimulation in spring when woodchucks usually arouse ending their hibernating season (Krupp et al. 1977; Nève and Dumont 1970, Wetzel, 1972). Within the cell, T_3_ is the most bioactive form of THs, and is usually generated by intracellular conversion from T_4_ via deiodinase enzymes (D2) (Fig. 4, ⑥). When intranuclear T_3_ receptors are present (Fig. 4, ⑦), T_3_ can act as transcription factor (van der Spek et al. 2017). Intriguingly, an increasing activity of the 5'-monodeiodinating enzyme that converts T_4_ into T_3_ was observed in the liver of newborn piglets until day 3 postnatally, accompanied by an increase of serum T_3_ (Brzezińska-Slebodzińska and Slebodziński 1986). And Magnus and Henderson measured higher hepatic nuclear T_3_ receptor concentrations in active than in dormant hibernators (Magnus and Henderson 1988b). Once the increased amount of freely circulating THs in the blood stream – influenced by the reduced binding affinity to serum proteins (TBG) – enters target cells via membrane transporters, T_3_ binds to its nuclear receptor and target proteins are synthesized, mostly mediating metabolic changes. The modality of these metabolic changes is mainly dependent on the effector organ itself (Fig. 4, ⑧) as well as the local TH bioavailability. For example, a low TH bioavailability, locally limited to the hypothalamus, can enable physiological anorexia in the hibernating ground squirrel without affecting peripheral TH concentrations (Mohr, 2024). Likewise, patients with a clinical manifestation of resistance to TH syndrome may experience hypo- to hyperthyroid symptoms simultaneously, owing to differing TH bioavailability in different target tissues (Zhao, 2023). Consequently, the quantification of circulating TH concentrations is insufficient to serve as an accurate indicator neither for their bioavailability in target tissues nor for their triggered actions in effector organs. Therefore, it is currently suggested to use indices, such as the thyrotropin-T_4_ resistance index (TT4RI), the TSH index (TSHI), the thyroid feedback quantile-based index (TFQI), or the serum fT_3_/fT_4_ ratio, to reflect central or peripheral TH bioavailability more accurately (Zhao, 2023).

### Metabolic effects of thyroid hormones upon arousal and at birth

#### Typical effector organs

A cooperative interaction of TH effector organs allows the rapid increase in MR with the initiation of thermoregulatory mechanisms as an integral characteristic of arousal and birth. Typical TH effector organs are the lung, the heart, the liver, fat tissue, in particular BAT, and the central nervous system (Fig. 4, ⑧) (Potenza et al. 2009)*.*

For long it has been known that THs generate heat to stabilize T_*b*_ by stimulating non-shivering thermogenesis in BAT by activating uncoupling proteins in mitochondria so the energy created by the proton gradient can dissipate as heat (Cannon and Nedergaard 2004; Bank et al. 2015). In the liver, THs promote catabolic processes like lipolysis and gluconeogenesis to provide energy substrates during these energy consuming processes (Sinha et al. 2014). In the heart and lung, THs increase the amount of catecholamine receptors to upregulate heart and breathing rate and promote lung fluid absorption at birth (Fowden and Forhead 2009; Graves and Haley 2013). Furthermore, it has been known for a long time that the neuronal development of the newborn is dependent on the presence of sufficient THs already during the intrauterine period as the clinical presentation of neonatal hypothyroidism exemplifies (Patel et al. 2011). Similarly, a positive correlation between TH presence and neuronal activity was found in ground squirrels during the non-hibernating period (Wilsterman et al. 2015). Another TH effect on the central nervous system was demonstrated in a recent study when local infusion of T_3_ in the hypothalamus of hibernating ground squirrels led to the reversal of hibernation-induced anorexia (Mohr, 2024).

#### Synergistic interactions with other endocrine factors

The TH axis triggers these physiological changes not exclusively. Other endocrine factors, for one the sympathetic nervous system, are deeply intertwined with the TH axis in a synergistic network (Fig. 4, ⑨). Concomitant to the surge of TSH and THs, a major boost in hormonal activity of cortisol and catecholamines can be detected in the hibernator upon arousal and in the newborn upon delivery (Fowden and Forhead 2009). For instance, at the stress of birth the burst in catecholamines, in particular epinephrine, can stimulate even higher TH availability (Graves and Haley 2013). And vice versa, THs are able to activate the sympathetic nervous system as well (Silva and Larsen 1983). For example, T_3_ sensitizes the sympathetic nervous system to activate uncoupling proteins in mitochondria of BAT to promote non-shivering thermogenesis (Bianco and Silva 1987a,b; Yen 2001; Silva and Larsen 1986). Not surprisingly, the potency of noradrenaline to increase heat production in BAT is diminished in hypothyroid animals (Ikemoto et al. 1967). This synergistic interaction of the HPT-axis with the sympathetic nervous system (Ortiga-Carvalho et al. 2016) has also been described to intensify upon the time of arousal when an increase in thermogenic capacity is pivotal to support thermogenesis for the rewarming process (Frare et al. 2018). Analogously, THs stimulate the pulmonary fluid absorption system in the neonate at birth by regulating its sensitivity to catecholamines and by an increase in amount and activity of pulmonary Na^+^-K^+^-ATPases (Barker et al. 1988, 1990; Ramminger et al. 2002). The ability of epinephrine and cAMP to switch from lung liquid secretion to absorption increases progressively towards term (Barker et al. 1988). But the epinephrine-induced lung liquid absorption not only requires T_3_ but also cortisol (Barker et al. 1990; Ramminger et al. 2002).

Accordingly, the regulation of TH bioavailability and action underlies a finely tuned feedback control system with numerous cofactors (Little 2016). Such possible cofactors that interact with the TH system may comprise environmental factors, such as T_*a*_, endogenous rhythm like photoperiodism (Reed 1995; Dardente et al. 2014), the placenta as an immunological barrier (Lieutaud 1999), and also still unidentified factors (Fig. 4, ⑨). So, in order to get a more accurate picture it is to emphasize that for the realistic evaluation of the TH system the interaction with other endocrine systems *and* the physiological context need to be taken into account (Little 2016). But even though studies on the TH system are innumerable, a comprehensive investigation including all regulatory components is yet to undertake (Little 2016).

### Difference between arousing from hibernation and being born

It appears that the TH axis is one important instrument in the endocrine ensemble that orchestrates the outstandingly well-coordinated phenomena of mammalian birth and arousal from hibernation. Identifying the underlying mechanisms of both transitional processes may also contribute to our understanding of selected medical issues (e.g., regulation of body weight or reversibility of renal failure) and even provide new approaches for favourably modulating MR during manned interplanetary space missions (Singer 2006; Choukèr et al. 2019).

Yet, it must be clarified that the comparative approach should not imply the equation of both phenomena. A fundamental difference is that the arousal process implies a rapid re-activation of previously decelerated physiological functions whereas cord-clamping at birth marks an abrupt initiation of physiological independence for the newborn. One is a survival strategy in response to adverse external conditions, the other is an endogenous maturation process resulting in physiological autonomy. Whether birth can be regarded as the first arousal from endogenous metabolic suppression or whether the state of hibernation can be deemed as a return to prenatal, intrauterine conditions remains open (Singer 2023).

But matter of fact is that endogenous metabolic suppression is a wide-spread ability and far beyond an atavistic survival strategy as it is seen in a wide range of small and large mammals including primate species (Geiser 2004; Heldmaier et al. 2004; Dausmann et al. 2004). A careful distinction between different types of hibernators (e.g., food-storing vs. fat-storing species) and the degree of neonatal maturity at birth as well as the miscellaneous patterns of metabolic suppression (torpor, hibernation, estivation, and others) is mandatory.

### Methodological constraints

The comparative analysis of the thyroid's role in mammalian birth and hibernation, or more specifically, arousal from hibernation, is subject to methodological constraints. For one, the number of publications measuring TRH, TSH, T_4_, T_3_, and rT_3_ dynamics around the time of arousal and at birth differ significantly, so this study widened inclusion criteria for the time frame of arousal from hibernation. In fact, the timing of material collection around the time of arousal at the end of hibernating season varies in the included studies of mammalian hibernators. Most of the included studies examined TH levels either throughout the annual cycle (e.g., Magnus and Henderson 1988a) or before and during hibernation and after arousal (e.g., Young et al. 1979b). However, the included studies specified neither a distinct time point of material collection, rather a time frame, nor was the moment of arousal stated more precisely. Consequently, there is a systematical divergence of the point in time of blood collection surrounding the arousal process from the very precise sample time point at the moment of birth. Secondly, varying technical methods in the quantification of serum TRH, TSH, T_4_, T_3_, and rT_3_ concentrations (RIA, equilibrium dialysis, electrochemiluminescence immunoassays) that were used in different studies may have an impact on the quality of the comparison. Another limiting fact is that some publications measured total and free T_4_ and T_3_ and a few exclusively total T_4_, T_3_ serum levels.

## Conclusion

Overall, the thorough comparison of mammalian birth versus arousal from hibernation reveals that although THs play a crucial role in both transitional phenomena, the postnatal onset of thyroid activity differs from the sustained readiness for arousal governing mammalian hibernation. At birth, the mammalian neonate is flooded with THs from the thyroid gland into the bloodstream, leading to a transitory “physiological hyperthyroidism”. Upon arousal, the mammalian hibernator can revive previously silenced TH activity through (a) reduced affinity to TBG, (b) presence of membrane transporters, and (c) activated deiodinase enzymes in target tissues. Neither arousal from hibernation nor mammalian birth are exclusively controlled by THs. However, both metabolic transitions are strongly supported by the THs’ stimulating effects on (a) catabolic pathways, (b) NST and (c) heart and breathing rates. Yet, THs exert these effects within a broader endocrine framework, particularly catecholamines and glucocorticoids, interrelated with their rate of conversion into biologically active forms in target tissues. Thus, an analysis of TH turnover rates in target tissues rather than the quantification of serum levels appears to reflect TH action more accurately.

## Supplementary Information

Below is the link to the electronic supplementary material.

Supplementary file1 (DOCX 29 KB)

## Data Availability

All data generated or analysed during this study are included in this published article. Additional citations for Tables 1, 2, 3 that are not referred to in the main reference list are provided in a supplementary file.
