# Supplementary material for: Mammalian birth versus arousal from hibernation: thyroid hormones, common regulators of metabolic transition?
Source: J Comp Physiol B. 2025 Apr 10;195(3):277–92. doi: 10.1007/s00360-025-01611-6 (PMC12289816; doi:10.1007/s00360-025-01611-6)
Supplement: Supplementary file 1 — Supplementary file1 (DOCX 29 KB) [file 360_2025_1611_MOESM1_ESM.docx]

**Supplementary Material**

to

Melanie Heidkamp, Annika Herwig, Dominique Singer:

Mammalian Birth versus Arousal from Hibernation –

Thyroid Hormones, Common Regulators of Metabolic Transition?

**References on the dynamics of TRH, TSH, T_4_, T_3_, and rT_3_ concentrations in mammalian hibernators upon (final) arousal (Table 1)**

AZIZI, F., MANNIX, J. E., HOWARD, D. & NELSON, R. A. 1979. Effect of winter sleep on pituitary-thyroid axis in American black bear. *Am J Physiol,* 237**,** E227-30.

BAUMAN, T. R., ANDERSON, R. R. & TURNER, C. W. 1968. Thyroid hormone secretion rates and food consumption of the hamster (Mesocricetus auratus) at 25.5 degree and 4.5 degree C. *Gen Comp Endocrinol,* 10**,** 92-8.

DAMASSA, D. A., GUSTAFSON, A. W., KWIECINSKI, G. G. & GAGIN, G. A. 1995. Seasonal influences on the control of plasma sex hormone-binding globulin by T4 in male little brown bats. *Am J Physiol,* 268**,** R1303-9.

DEMENEIX, B. A. & HENDERSON, N. E. 1978a. Serum T4 and T3 in active and torpid ground squirrels, Spermophilus richardsoni. *Gen Comp Endocrinol,* 35**,** 77-85.

DEMENEIX, B. A. & HENDERSON, N. E. 1978b. Thyroxine metabolism in active and torpid ground squirrels, Spermophilus richardsoni. *Gen Comp Endocrinol,* 35**,** 86-92.

HULBERT, A. J. & HUDSON, J. W. 1976. Thyroid function in a hibernator, Spermophilus tridecemlineatus. *Am J Physiol,* 230**,** 1211-6.

KWIECINSKI, G. G., DAMASSA, D. A. & GUSTAFSON, A. W. 1991. Patterns of plasma sex hormone-binding globulin, thyroxine and thyroxine-binding globulin in relation to reproductive state and hibernation in female little brown bats. *J Endocrinol,* 128**,** 63-70.

MAGNUS, T. H. & HENDERSON, N. E. 1988a. Thyroid hormone resistance in hibernating ground squirrels, Spermophilus richardsoni. I. Increased binding of triiodo-L-thyronine and L-thyroxine by serum proteins. *Gen Comp Endocrinol,* 69**,** 352-60.

MAGNUS, T. H. & HENDERSON, N. E. 1988b. Thyroid hormone resistance in hibernating ground squirrels, Spermophilus richardsoni. II. Reduction of hepatic nuclear receptors. *Gen Comp Endocrinol,* 69**,** 361-71.

MCCAIN, S., RAMSAY, E. & KIRK, C. 2013. The effects of hibernation and captivity on glucose metabolism and thyroid hormones in American black bear (Ursus americanus). *J Zoo Wildl Med,* 44**,** 324-32.

NELSON, R. A. 1973. Winter sleep in the black bear. A physiologic and metabolic marvel. *Mayo Clin Proc,* 48**,** 733-7.

NELSON, R. A., WAHNER, H. W., JONES, J. D., ELLEFSON, R. D. & ZOLLMAN, P. E. 1973. Metabolism of bears before, during, and after winter sleep. *Am J Physiol,* 224**,** 491-6.

NEVRETDINOVA, Z., SOLOVENCHUK, L. & LAPINSKI, A. 1992. Some aspects of lipid metabolism and thyroid function in arctic ground squirrel, Citellus parryi during hibernation. *Arctic Med Res,* 51**,** 196-204.

NICOL, S. C., ANDERSEN, N. A. & TOMASI, T. E. 2000. Seasonal variations in thyroid hormone levels in free-living echidnas (Tachyglossus aculeatus). *Gen Comp Endocrinol,* 117**,** 1-7.

RICHARDSON, C. S., HEEREN, T. & KUNZ, T. H. 2018. Seasonal and Sexual Variation in Metabolism, Thermoregulation, and Hormones in the Big Brown Bat (Eptesicus fuscus). *Physiol Biochem Zool,* 91**,** 705-715.

TOMASI, T. E., HELLGREN, E. C. & TUCKER, T. J. 1998. Thyroid hormone concentrations in black bears (Ursus americanus): hibernation and pregnancy effects. *Gen Comp Endocrinol,* 109**,** 192-9.

WILSTERMAN, K., BUCK, C. L., BARNES, B. M. & WILLIAMS, C. T. 2015. Energy regulation in context: Free-living female arctic ground squirrels modulate the relationship between thyroid hormones and activity among life history stages. *Horm Behav,* 75**,** 111-9.

YOUNG, R. A., DANFORTH, E., JR., VAGENAKIS, A. G., KRUPP, P. P., FRINK, R. & SIMS, E. A. 1979a. Seasonal variation and the influence of body temperature on plasma concentrations and binding of thyroxine and triiodothyronine in the woodchuck. *Endocrinology,* 104**,** 996-9.

YOUNG, R. A., ROBINSON, D. S., VAGENAKIS, A. G., SAAVEDRA, J. M., LOVENBERG, W., KRUPP, P. P. & DANFORTH, E., JR. 1979b. Brain TRH, monoamines, tyrosine hydoxylase, and tryptophan hydroxylase in the woodchuck, Maromota monax, during the hibernation season. *Comp Biochem Physiol C Comp Pharmacol,* 63c**,** 319-23.

**References on the dynamics of TRH, TSH, T_4_, T_3_, and rT_3_ concentrations in human neonates (Table 2)**

ABUID, J., KLEIN, A. H., FOLEY, T. P., JR. & LARSEN, P. R. 1974. Total and free triiodothyronine and thyroxine in early infancy. *J Clin Endocrinol Metab,* 39**,** 263-8.

ABUID, J., STINSON, D. A. & LARSEN, P. R. 1973. Serum triiodothyronine and thyroxine in the neonate and the acute increases in these hormones following delivery. *J Clin Invest,* 52**,** 1195-9.

CAVALLO, L., MARGIOTTA, W. & KERNKAMP, C. 1978. Serum concentrations of TSH, T4, and T3 during the first three days of postnatal life. *Boll Soc Ital Biol Sper,* 54**,** 1099-103.

CAVALLO, L., MARGIOTTA, W., KERNKAMP, C. & PUGLIESE, G. 1980. Serum levels of thyrotropin, thyroxine, 3,3',5-triiodothyronine and 3,3',5'-triiodothyronine (reverse T3) in the first six days of life. *Acta Paediatr Scand,* 69**,** 43-7.

CHEN, Y., SHEN, T., HE, Y., CHEN, X., CHEN, D. 2024. Association between maternal age and sex-based neonatal free triiodothyronine levels. *BMC Endocr Disord* 24(1): 98.

CHOPRA, I. J. 1974. A radioimmunoassay for measurement of 3,3',5'-triiodothyronine (reverse T3). *J Clin Invest,* 54**,** 583-92.

CHOPRA, I. J., SACK, J. & FISHER, D. A. 1975. Circulating 3,3', 5'-triiodothyronine (reverse T3) in the human newborn. *J Clin Invest,* 55**,** 1137-41.

CZERNICHOW, P., GREENBERG, A. H., TYSON, J. & BLIZZARD, R. M. 1971. Thyroid Function Studied in Paired Maternal-Cord Sera and Sequential Observations of Thyrotropic Hormone Release during the First 72 Hours of Life. *Pediatric Research,* 5**,** 53-58.

ERENBERG, A., PHELPS, D. L., LAM, R. & FISHER, D. A. 1974. Total and free thyroid hormone concentrations in the neonatal period. *Pediatrics,* 53**,** 211-6.

FISHER, D. A., DUSSAULT, J. H., SACK, J. & CHOPRA, I. J. 1976. Ontogenesis of hypothalamic--pituitary--thyroid function and metabolism in man, sheep, and rat. *Recent Prog Horm Res,* 33**,** 59-116.

FISHER, D. A., NELSON, J. C., CARLTON, E. I. & WILCOX, R. B. 2000. Maturation of human hypothalamic-pituitary-thyroid function and control. *Thyroid,* 10**,** 229-34.

FISHER, D. A. & ODELL, W. D. 1969. Acute release of thyrotropin in the newborn. *J Clin Invest,* 48**,** 1670-7.

FISHER, D. A., ODELL, W. D., HOBEL, C. J. & GARZA, R. 1969. Thyroid function in the term fetus. *Pediatrics,* 44**,** 526-35.

GEIGER, W. 1973. Radioimmunological determination of human chorionic gonadotropin, human placental lactogen, growth hormone and thyrotropin in the serum of mother and child during the early puerperium. *Horm Metab Res,* 5**,** 342-6.

HOMOKI, J., BIRK, J., LOOS, U., ROTHENBUCHNER, G., FAZEKAS, A. T. & TELLER, W. M. 1975. Thyroid function in term newborn infants with congenital goiter. *J Pediatr,* 86**,** 753-8.

HÜFNER, M., HESCH, R. D., HEINRICH, U. & LÜDERS, D. 1973. Plasma triiodothyronine at the end of pregnancy, in the cord blood and in the first days of the newborn. *Z Kinderheilkd,* 114**,** 301-4.

JACOBSEN, B. B., ANDERSEN, H. J., PEITERSEN, A. B., DIGE-PETERSEN, H. & HUMMER, L. 1977. Serum levels of thyrotropin, thyroxine and triiodothyronine in fullterm, small-for-gestational age and preterm newborn babies. *Acta Paediatr Scand,* 66**,** 681-7.

KLEIN, A. H., ODDIE, T. H., PARSLOW, M., FOLEY, T. P., JR. & FISHER, D. A. 1982. Developmental changes in pituitary-thyroid function in the human fetus and newborn. *Early Hum Dev,* 6**,** 321-30.

KNOBEL, R. B. 2007. Thyroid hormone levels in term and preterm neonates. *Neonatal Netw,* 26**,** 253-9.

KRATZSCH, J. & PULZER, F. 2008. Thyroid gland development and defects. *Best Pract Res Clin Endocrinol Metab,* 22**,** 57-75.

LEMARCHAND-BÉRAUD, T., GENAZZANI, A. R., BAGNOLI, F. & CASOLI, M. 1972. Thyroid function in the premature and the full term newborn. *Acta Endocrinol (Copenh),* 70**,** 445-53.

LOMBARDI, G., LUPOLI, G., SCOPACASA, F., PANZA, R. & MINOZZI, M. 1978. Plasma immunoreactive thyrotropin releasing hormone (TRH) values in normal newborns. *J Endocrinol Invest,* 1**,** 69-72.

MONTALVO, J. M., WAHNER, H. W., MAYBERRY, W. E. & LUM, R. K. 1973. Serum triiodothyronine, total thyroxine, and thyroxine to triiodothyronine ratios in paired maternal-cord sera and at one week and one month of age. *Pediatr Res,* 7**,** 706-11.

MUTLU, M., KARAGÜZEL, G., ALıYAZICIOĞLU, Y., EYÜPOĞLU, I., OKTEN, A. & ASLAN, Y. 2012. Reference intervals for thyrotropin and thyroid hormones and ultrasonographic thyroid volume during the neonatal period. *J Matern Fetal Neonatal Med,* 25**,** 120-4.

ODDIE, T. H., BERNARD, B., PRESLEY, M., KLEIN, A. H. & FISHER, D. A. 1978. Damped oscillations in serum thyroid hormone levels of normal newborn infants. *J Clin Endocrinol Metab,* 47**,** 61-5.

ODELL, W. D., WILBER, J. F. & UTIGER, R. D. 1967. Studies of thyrotropin physiology by means of radioimmunoassay. *Recent Prog Horm Res,* 23**,** 47-85.

PEZZINO, V., FILETTI, S., BELFIORE, A., PROTO, S., DONZELLI, G. & VIGNERI, R. 1981. Serum thyroglobulin levels in the newborn. *J Clin Endocrinol Metab,* 52**,** 364-6.

PICKERING, D. E., KONTAXIS, N. E., BENSON, R. C. & MEECHAN, R. J. 1958. Thyroid function in the perinatal period. *AMA J Dis Child,* 95**,** 616-21.

POLAK, M. & LUTON, D. 2014. Fetal thyroïdology. *Best Pract Res Clin Endocrinol Metab,* 28**,** 161-73.

ROGOWSKI, P., SIERSBAEK-NIELSEN, K. & HANSEN, J. M. 1974. Estimation of free thyroxine index in the newborn using micro-methods. *Acta Paediatr Scand,* 63**,** 201-4.

SACK, J., FISHER, D. A. & WANG, C. C. 1976. Serum thyrotropin, prolactin, and growth hormone levels during the early neonatal period in the human infant. *J Pediatr,* 89**,** 298-300.

SANTINI, F., CHIOVATO, L., GHIRRI, P., LAPI, P., MAMMOLI, C., MONTANELLI, L., SCARTABELLI, G., CECCARINI, G., COCCOLI, L., CHOPRA, I. J., BOLDRINI, A. & PINCHERA, A. 1999. Serum iodothyronines in the human fetus and the newborn: evidence for an important role of placenta in fetal thyroid hormone homeostasis. *J Clin Endocrinol Metab,* 84**,** 493-8.

SIMILÄ, S., KOIVISTO, M., RANTA, T., LEPPÄLUOTO, J., REINILÄ, M. & HAAPALAHTI, J. 1975. Serum tri-iodothyronine, thyroxine, and thyrotrophin concentrations in newborns during the first 2 days of life. *Arch Dis Child,* 50**,** 565-7.

STUBBE, P., GATZ, J., HEIDEMANN, P., MÜHLEN, A. & HESCH, R. 1978. Thyroxine-binding globulin, triiodothyronine, thyroxine and thyrotropin in newborn infants and children. *Horm Metab Res,* 10**,** 58-61.

TOUZERY, C., PELLETIER, J. L., GAILLIARD, P., TENENBAUM, D., BRESARD, B., MICHIELS, Y., ALISON, M. & NIVELON, J. L. 1978. [Thyroxine: normal levels from birth to 70 days. Value for the detection of congenital hypothyroidism]. *Nouv Presse Med,* 7**,** 2965.

UTIGER, WILBER, CORNBLATH, HARM & MACK 1968. TSH Secretion in Newborn Infants and Children. *The Journal of Clinical Investigation,* 47**,** 97a - 98a.

YAMAZAKI, E., NOGUCHI, A. & SLINGERLAND, D. W. 1961. Thyrotropin in the serum of mother and fetus. *J Clin Endocrinol Metab,* 21**,** 1013-4.

**References on the dynamics of TRH, TSH, T_4_, T_3_, and rT_3_ concentrations in non-human mammals at birth (Table 3)**

BRZEZIŃSKA-SLEBODZIŃSKA, E. & SLEBODZIŃSKI, A. B. 1986. Simultaneous observations of iodothyronine content in the thyroid gland, serum and thyroxine 5'- and 5-monodeiodinase activity in liver, during the neonatal period of the pig. *J Dev Physiol,* 8**,** 79-86.

CABELLO, G. & LEVIEUX, D. 1980. Neonatal changes in the concentrations of thyrotropin, triiodothyronine, thyroxine and cortisol in the plasma of pre-term and full-term lambs. *J Dev Physiol,* 2**,** 59-69.

CABELLO, G. & LEVIEUX, D. 1981. Hormonal status in the newborn lamb (cortisol, T3, T4). Relationships to the birth weight and the length of gestation: effect of the litter size. *Biol Neonate,* 39**,** 208-16.

CABELLO, G. & WRUTNIAK, C. 1986. Plasma free and total iodothyronine levels in the newborn lamb. Physiological considerations. *Reprod Nutr Dev,* 26**,** 1281-8.

CABELLO, G. & WRUTNIAK, C. 1989. Thyroid hormone and growth: relationships with growth hormone effects and regulation. *Reprod Nutr Dev,* 29**,** 387-402.

CABELLO, G. & WRUTNIAK, C. 1990. Thyroid function in the newborn lamb. Physiological approach of the mechanisms inducing the changes in plasma thyroxine, free thyroxine and triiodothyronine concentrations. *J Dev Physiol,* 13**,** 25-32.

DAVICCO, M. J., LEFAIVRE, J. & BARLET, J. P. 1982a. Plasma iodothyronine levels in lambs during the perinatal period: influence of thyrotropin injection. *Reprod Nutr Dev,* 22**,** 557-67.

DAVICCO, M. J., VIGOUROUX, E., DARDILLAT, C. & BARLET, J. P. 1982b. Thyroxine, triiodothyronine and iodide in different breeds of newborn calves. *Reprod Nutr Dev,* 22**,** 355-62.

DUSSAULT, J. H. & LABRIE, F. 1975. Development of the hypothalamic-pituitary-thyroid axis in the neonatal rat. *Endocrinology,* 97**,** 1321-4.

ENGELHARDT, F. R. & FERGUSON, J. M. 1980. Adaptive hormone changes in harp seals, Phoca groenlandica, and gray seals, Halichoerus grypus, during the postnatal period. *Gen Comp Endocrinol,* 40**,** 434-45.

FISHER, D. A., DUSSAULT, J. H., SACK, J. & CHOPRA, I. J. 1976. Ontogenesis of hypothalamic--pituitary--thyroid function and metabolism in man, sheep, and rat. *Recent Prog Horm Res,* 33**,** 59-116.

GRÜNBERG, W., STEINHARDT, M., RATH, D. & NIEMANN, H. 1998. Thyroid hormones in Old German Black Pied- and Holstein-Friesian suckling calves. Effects of parturition and postpartum adaptation. *Tierarztl Prax Ausg G Grosstiere Nutztiere,* 26**,** 318-25.

HABIBU, B., UMARU KAWU, M., ALUWONG, T., JOAN MAKUN, H. 2022. Neonatal thermoregulation and dynamics of serum thyroid hormones in tropical breeds of goat kids. *J Therm Biol* 108: 103299.

HAULENA, M., AUBIN, D. J. & DUIGNAN, P. J. 1998. Thyroid hormone dynamics during the nursing period in harbour seals, Phoca vitulina. *Canadian Journal of Zoology,* 76**,** 48-55.

HERNANDEZ, M. V., ETTA, K. M., REINEKE, E. P., OXENDER, W. D. & HAFS, H. D. 1972. Thyroid function in the prenatal and neonatal bovine. *J Anim Sci,* 34**,** 780-5.

IRVINE, C. H. & EVANS, M. J. 1975. Postnatal changes in total and free thyroxine and triiodothyronine in foal serum. *J Reprod Fertil Suppl***,** 709-15.

KAHL, S., WRENN, T. R. & BITMAN, J. 1977. Plasma tri-iodothyronine and thyroxine in young growing calves. *J Endocrinol,* 73**,** 397-8.

KIEFFER, J. D., MOVER, H., FEDERICO, P. & MALOOF, F. 1976. Pituitary-thyroid axis in neonatal and adult rats: comparison of the sexes. *Endocrinology,* 98**,** 295-304.

KLEIN, A. H., ODDIE, T. H. & FISHER, D. A. 1978. Effect of parturition on serum iodothyronine concentrations in fetal sheep. *Endocrinology,* 103**,** 1453-7.

KLEIN, A. H., ODDIE, T. H. & FISHER, D. A. 1980. Iodothyronine kinetic studies in the newborn lamb. *J Dev Physiol,* 2**,** 29-35.

KÜHN, E. R., VAN OSSELAER, P., SIAU, O., DECUYPERE, E. & MOREELS, A. 1986. Thyroid function in newborn lambs: influence of prolactin and growth hormone. *J Endocrinol,* 109**,** 215-9.

LEATHERLAND, J. F. A. R., K. 1979. Thyroid activity in adult and neonate harp seals Pagophilus groenlandicus. *Journal of Zoology (London),* 189**,** 399-405.

LITTLE, G. J. 1991. Thyroid morphology and function and its role in thermoregulation in the newborn southern elephant seal (Mirounga leonina) at Macquarie Island. *J Anat,* 176**,** 55-69.

MATHUR, H., BROWN, B. L., KRANE, E. J., THOMAS, A. L. & NATHANIELSZ, P. W. 1980. Thyroid hormone relationships in the fetal and newborn lamb. *Biol Neonate,* 37**,** 138-41.

NATHANIELSZ, P. W. 1969. Plasma thyroxine levels in the young lamb from birth to 61 days. *J Endocrinol,* 45**,** 475-6.

NATHANIELSZ, P. W. 1975. Thyroid function in the fetus and newborn mammal. *Br Med Bull,* 31**,** 51-6.

NATHANIELSZ, P. W., SILVER, M. & COMLINE, R. S. 1973. Plasma tri-iodothyronine concentration in the foetal and newborn lamb. *J Endocrinol,* 58**,** 683-4.

NATHANIELSZ, P. W. & THOMAS, A. L. 1973. Plasma triiodothyronine concentration in the newborn calf. *Experientia,* 29**,** 1426-1426.

NOWAK, G. 1983. Free thyroid hormone levels during the postnatal period in the pig. *Biol Neonate,* 43**,** 164-71.

PALS, A. J., REINEKE, E. P. & SHAW, G. H. 1973. Serum thyroxine levels in the perinatal guinea pig (Cavia porcellus). *Lab Anim Sci,* 23**,** 511-4.

SACK, J., BEAUDRY, M., DELAMATER, P. V., OH, W. & FISHER, D. A. 1976. Umbilical cord cutting triggers hypertriiodothyroninemia and nonshivering thermogenesis in the newborn lamb. *Pediatr Res,* 10**,** 169.

SLEBODZIŃSKI, A. 1971. Serum free thyroxine levels in newborn animals assessed by the sephadex tri-iodothyronine-binding coefficient. *J Endocrinol,* 50**,** 349-50.

SLEBODZIŃSKI, A. B. & COGIEL, F. 1983. Serum thyroid hormone levels in colostrum deprived piglets and calves. *Endocrinol Exp,* 17**,** 263-70.

SLEBODZIŃSKI, A. B., NOWAK, G. & ZAMYSLOWSKA, H. 1981. Sequential observation of changes in thyroxine, triiodothyronine and reverse triiodothyronine during the postnatal adaptation of the pig. *Biol Neonate,* 39**,** 191-9.

STEINHARDT, M., THIELSCHER, H. H., VON HORN, T., VON HORN, R., ERMGASSEN, K. & SMIDT, D. 1996. [Thyroid hormones in dairy calves after birth and in the first days of life. Amplitude of variation, maternal-fetal relationships and reaction patterns specific to the individual]. *Dtsch Tierarztl Wochenschr,* 103**,** 136-41.

STOKKAN, K. A., VAUGHAN, M. K., REITER, R. J., FOLKOW, L. P., MÅRTENSSON, P. E., SAGER, G., LYDERSEN, C. & BLIX, A. S. 1995. Pineal and thyroid functions in newborn seals. *Gen Comp Endocrinol,* 98**,** 321-31.

TAKAHASHI, K., TAKAHASHI, E., DUCUSIN, R. J., TANABE, S., UZUKA, Y. & SARASHINA, T. 2001. Changes in serum thyroid hormone levels in newborn calves as a diagnostic index of endemic goiter. *J Vet Med Sci,* 63**,** 175-8.

WOLDSTAD, S. & JENSSEN, B. M. 1999. Thyroid hormones in grey seal pups (Halichoerus grypus). *Comp Biochem Physiol A Mol Integr Physiol,* 122**,** 157-62.

WRUTNIAK, C. & CABELLO, G. 1987. Neonatal changes in plasma cortisol, free and total iodothyronine levels in control and hypotrophic lambs. *Reprod Nutr Dev,* 27**,** 945-53.
